# Understanding clozapine-related blood dyscrasias. Developments, genetics, ethnicity and disparity: it's a CIN

**DOI:** 10.1192/bjb.2024.38

**Published:** 2025-06

**Authors:** Edward Silva, Sophie Legge, Cecilia Casetta, Eromona Whiskey, Ebenezer Oloyede, Siobhan Gee

**Affiliations:** 1 Mersey Care NHS Foundation Trust, Liverpool, UK; 2 Cardiff University, Cardiff, UK; 3 South London and Maudsley NHS Foundation Trust, London, UK; 4 King's College London, London, UK; 5 University of Oxford, Oxford, UK

**Keywords:** Clozapine, schizophrenia, agranulocytosis, neutropenia, benign ethnic neutropenia

## Abstract

Clozapine remains the gold standard intervention for treatment-resistant schizophrenia; however, it remains underused, especially for some minority groups. A significant impediment is concern about propensity to neutropenia. The aim of this article is to provide an update on current knowledge relating to: the pattern and incidence of severe blood dyscrasias; the effectiveness of current monitoring regimes in reducing harm; the mechanisms of and the distinctions between clozapine-induced neutropenia and agranulocytosis; benign ethnic neutropenia; and changes to the monitoring thresholds in the USA and other international variations. These all have implications for the practical use of clozapine; specifically, how barriers to initiating, maintaining and restarting clozapine can be understood and in many cases overcome, especially for patients from minority groups, potentially with simpler approaches than the use of lithium or G-CSF.

After more than 50 years, clozapine still has a unique place in the treatment of schizophrenia. It remains the only treatment likely to work for the 30% or so who do not show an adequate response to one or more other antipsychotics.^1^ The increased risk of agranulocytosis was identified in the early 1970s shortly after its first use. Since then, risk management has involved regular full blood counts to monitor for falls in absolute neutrophil count (ANC). This has proved to be highly effective in avoiding harm from serious adverse events caused by blood dyscrasias. However, the drug remains underused. In the UK, fewer than a third of eligible patients receive clozapine, and when it is initiated this is usually after many years of suboptimal treatment with multiple antipsychotics.^2,3^ Patients from ethnic minorities are particularly disadvantaged.^4^ Despite the evidence of superior outcomes including not only symptomatic improvement and reduced hospital admissions but also patient preferences and overall mortality, prescribers remain reluctant to initiate clozapine, and concern about blood dyscrasias is a significant impediment.^5,6^ Over the past decade, our understanding of clozapine-related blood dyscrasias has developed and should be reassuring to patients and prescribers alike. This is important. There are significant adverse consequences to discontinuing clozapine. Relapse of psychosis is near-inevitable, and patients and carers describe having to stop clozapine owing to blood dyscrasia as being a stressful and worrying event.^7,8^ It is imperative that unnecessary treatment cessation is avoided.

## Blood monitoring

**Fig. 1:**
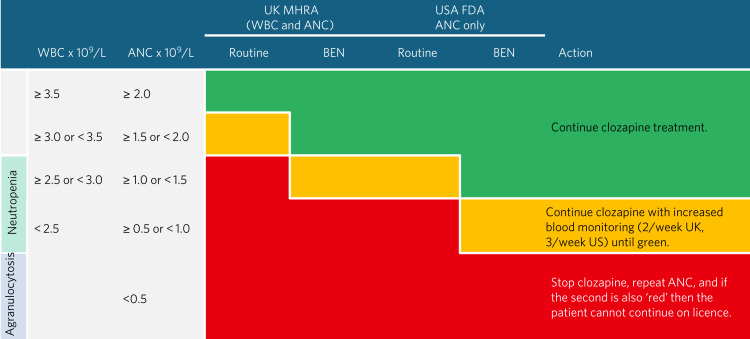
UK and US clozapine monitoring and risk management requirements. MHRA, Medicines and Healthcare Products Regulatory Authority; FDA, Food and Drug Administration; WBC, white blood cell count; ANC, absolute neutrophil count; BEN, benign ethnic neutropenia.

In 1975, nine cases of death from agranulocytosis were reported from Finland, weekly blood monitoring was introduced and, although Sandoz continued to manufacture clozapine for sale in Europe, further development halted until Kane's landmark study.^9^ Having been withdrawn in the UK, it was licensed again in early 1989, with compulsory blood count monitoring within stringent parameters, which were set with a wide margin of safety but arbitrarily.^10^ Both ANC and white cell counts are required, with action according to a traffic light system: green, carry on; amber, enhanced monitoring; and red, stop. The frequency of monitoring reduces from weekly to fortnightly after 18 weeks and then to every 4 weeks after 1 year. Patients recording two consecutive ‘red’ results are entered into the Central Non-Rechallenge Database (CNRD), which is shared between the three UK clozapine providers. These patients may not be prescribed clozapine within licence again. The US and UK thresholds for clozapine monitoring were identical until 2015, at which point the US Food and Drug Administration (FDA) approved a reduction of 0.5 × 10^−9^/L for each ANC range and removed the requirement for white blood cell, eosinophil and platelet monitoring completely. A retrospective analysis of a large cohort of veterans (*n* = 14 620) revealed that using the updated US criteria would have allowed significant numbers of patients with low baseline neutrophils to receive clozapine. Of those already taking clozapine in this group (*n* = 160), 14 had treatment interrupted owing to reductions in ANC – only two of these interruptions would have been necessary if the modified criteria had been followed.^11^ There are other international variations in the frequency, duration and administration of monitoring. In western Europe, most counties have weekly monitoring for 18 weeks, then every 4 weeks but without the company monitoring and pharmacy and prescriber registration used in the UK. The least restrictive regimes are in Iceland, where after 18 weeks bloods are taken four-monthly, and in The Netherlands, where monitoring can be stopped or reduced to three-monthly after 6 months of treatment, with no demonstrated increased risk to patients from either. This is unsurprising, given that monthly blood sampling is highly unlikely to detect the sudden and profound falls in ANC that accompany agranulocytosis.^12,13^ The UK and US regimes are set out in Fig. 1 and include those for people with benign ethnic neutropenia (BEN).

## Mechanisms, incidence and patterns of clozapine-induced agranulocytosis (CIA)

CIA is a type B idiosyncratic adverse drug reaction (ADR). It is unrelated to the mode of therapeutic action, and there is no evidence that it is dose dependent.^14^ Carbamazepine-induced severe adverse skin reactions are also type B ADRs and have predictive but different human leucocyte antigen (HLA) risk alleles for northern European and Han Chinese populations.^15,16^ Clozapine is one of many non-chemotherapy drugs that can cause idiosyncratic drug-induced agranulocytosis (IDIN), and approaches to risk management vary. The antithyroid drug carbimazole has a similar agranulocytosis risk (0.3–0.5%) and is used without mandatory blood monitoring.^17^ The mechanisms of CIA have not been fully elucidated but are thought to involve a combination of toxic, immunological and genetic factors, combined with oxidised drug metabolites and HLA-activating T helper cells, which induce B cells to produce drug-dependent neutrophil antibodies.^14^ Severe life-threatening CIA has a distinctive pattern, with a continuous and rapid fall to zero or near-zero ANC within 2–15 days, followed by a prolonged nadir of a similar duration.^13^ Genetic linkage studies have identified specific HLA types and transporter genes as conferring increased risk, but the pharmacogenetics has not yet progressed so as to make testing sufficiently predictive to be used clinically, and the identified genetic risk factors are not generalisable across ethnic groups.^18,19^ Overall, IDINs now have a mortality estimated at 5%; this is a marked improvement compared with previous levels, owing to early recognition and the introduction of hematopoietic growth factors such as G-CSF (granulocyte colony stimulating factor).^14^

Clozapine-induced neutropenia (CIN) and CIA are often regarded as synonymous, with the belief that CIN is a precursor to the more serious dyscrasia. However, there is little if any evidence to support this idea. Instead, it seems that the two are distinct, with most cases of CIN being incidental findings and artefacts of increased blood monitoring.

Although the incidence of CIA (0.9%) is well known, it is less well known that monitoring systems are highly effective in preventing sepsis and death from blood dyscrasias. When clozapine is used with blood monitoring, the incidence of death from agranulocytosis is 0.013%, or approximately 1 in 8000 patients prescribed clozapine.^20^ This is the metric that is of significance to clinicians and people who take clozapine, and it is what clinicians should explain when discussing the relative risks and benefits of treatment. Although it can be argued that it is the monitoring that results in what appears to be excellent risk mitigation, data from Iceland demonstrate an equal risk of neutropenia in patients taking clozapine and other antipsychotics, and meta-analysis of controlled trial data also fails to show that clozapine has a stronger association with neutropenia than other antipsychotic medications, or to find a difference in rates of agranulocytosis before and after 1990 (at which point mandatory monitoring was introduced).^21,22^ Further, Li et al did not find a lower incidence of agranulocytosis in studies with strict monitoring compared with those without.^23^

During clozapine treatment, the incidence of agranulocytosis peaks at 4 weeks; more than 80% of events occur within the first 18 weeks, and risk decreases markedly after this.^20^ During the risk period, the UK monitoring system is sensitive but not specific. After 18 weeks, utility decreases and risks identifying many patients as ineligible for clozapine in the first place, with many more false positives for clozapine-induced agranulocytosis risk after treatment has started. Monthly testing lacks a logical basis given the pattern of agranulocytosis and also has only a small chance of identifying a sudden and dramatic fall, and the clinical and economic utility of testing beyond 18 weeks is increasingly being questioned.^24,25^

ADR reporting for clozapine is extremely stringent. The Medicines and Healthcare Products Regulatory Authority (MHRA) figures show that to date there have been 8 deaths in the UK from clozapine induced agranulocytosis, one attributed to thrombocytopenia and none from other blood dyscrasias,^26^ clozapine has been licensed in the UK since 1989 and in the UK about 40 000 people currently take clozapine.^3^

## BEN, Duffy-null genotype, racial disparities in the use of clozapine and other normal neutrophil variability

What is a ‘normal’ neutrophil count? There is significant variation in people's baseline neutrophil count, and some individuals have a neutrophil count that is lower than two standard deviations below the mean from the UK's guidelines (calculated from individuals of White European ancestry) but is not pathological.^27,28^ This is crucial when considering access to clozapine. A lower-than-expected neutrophil count may be misinterpreted as pathological, resulting in access to the best available treatment being withheld. Minority groups frequently receive inferior healthcare, and access to clozapine is no exception; misunderstanding the significance of ethnicity and neutrophil counts is likely to be one of many reasons for this. In the UK, Black patients compared with others are half as likely to receive clozapine, even when multiple confounders are considered.^29^ Some minority groups are at increased risk of being unnecessarily excluded from clozapine treatment owing to their normal variation in neutrophil counts. BEN is the term currently used to describe non-pathological but persistent low levels of neutrophils in individuals with certain ethnicities. This is most common in individuals with African but also Middle Eastern, some Asian and other genetic ancestries. To label a non-pathological difference in baseline neutrophil levels in individuals who do not have European genetic ancestries a ‘condition’ is, of course, highly problematic.^30^ People identified with BEN have been found to have the expected haematopoietic stem cell numbers and myeloid maturation, do not suffer from more severe infections, and are not at increased risk of infection generally or when treated with drugs with a propensity to cause IDIN. The molecular cause of BEN is now known to be the Duffy-null genotype in the atypical chemokine receptor 1 (*ACKR1*) gene. This confers an evolutionary advantage by providing some resistance to malaria and has a frequency of up to 80% in Black African/Caribbean people in the UK and 65% of African Americans.^31,32^ The clinical significance of this extends beyond psychiatry; BEN has been implicated in disparities in survival between patients of African and European descent receiving chemotherapy, with non-pathological low neutrophil counts due to BEN resulting in delays, dose reductions and premature discontinuations of treatment.^33^ The UK clozapine providers all have provision to authorise reduced ANC and white blood cell count thresholds if a haematologist confirms BEN. The significance is that BEN is often unrecognised and can be burdensome to identify when suspected. This unnecessarily prevents clozapine initiation and can also result in mistaken withdrawal of clozapine in patients who have BEN that was not recognised at the time of clozapine initiation (an individual with BEN may have neutrophil counts that vary both above and below the normal range for White European ancestry populations). Even then, some individuals with BEN have pre-clozapine ANCs which are very low, or even below the adjusted UK thresholds, making those with BEN at greater risk of a fall in ANC that is sufficient to discontinue clozapine but which is not itself an ADR, rather a fluctuation within the normal range found in individuals with the Duffy-null genotype.^34,35^ Further, some individuals may have low baseline ANCs but not have BEN, and these people are also at risk of exclusion from clozapine initiation or unnecessary discontinuation of treatment. It is useful to know that in normal individuals the numbers of circulating neutrophils can vary without changes in the total number in the body. Circulating neutrophils increase in the afternoon and evening, after exercise and smoking.^36^

## Significance for UK psychiatrists

Knowledge of the probable mechanisms of CIA, the significance of the timing of an abnormal finding in relation to the duration of clozapine treatment, the time of day of blood sampling and the safety of the revised US thresholds is useful for all those involved in the drug treatment of psychosis. It can be used to put risk in context when making prescribing decisions and to fully explain the relative risks and benefits of clozapine to patients, their families and team members alike.

Clozapine is still the best treatment after clozapine has been withdrawn, and some people with treatment-resistant schizophrenia experience severe distress, prolonged hospital admission and, in some forensic cases, management with very prolonged seclusion. Restarting after neutropenia may be the only therapeutic option that is likely to have a significant effect.^37^ However, using clozapine after an episode of agranulocytosis (as opposed to neutropenia) demonstrably attributed to clozapine is not recommended and risks another severe episode.

In the UK, people of Black African and Caribbean heritage are more likely to be detained in hospital, have longer lengths of stay and higher rates of readmission and seclusion, are less likely to be offered or complete psychological therapies for psychosis and are much less likely to be prescribed clozapine than their White peers. There may be many contributing factors to the disproportionate underuse of clozapine in this group.^29,38^ Knowledge of the variation in normal biology among ethnic groups is essential to ensure that the best available treatments are used equitably. Many patients from Black and other minority backgrounds are likely to be mistakenly considered ineligible for clozapine and at increased risk of blood dyscrasias, and their treatment is less likely to be recommenced owing to unrecognised BEN. Many others may have successfully been treated with clozapine but then had this unnecessarily withdrawn following a ‘low’ neutrophil count due to unrecognised BEN. This was a finding from two UK inner-city trusts in Birmingham and South London. Of the 18 Black patients for whom clozapine had been prohibited, a retrospective review found that eight had BEN and, had the BEN criteria been used from initiation, none would have had clozapine withheld.^35^ Obtaining a haematological confirmation of BEN status is best achieved with a focused referral setting out ethnicity, repeated ANC counts below the laboratory normal range in the absence of infection, a physical examination and some baseline tests. Self-reported ethnicity can be supplemented by asking for the ethnicity of grandparents. Although many hospitals will be able to find a local haematologist, if this proves difficult then all the UK clozapine providers have contactable haematologists familiar with the issues and able to provide assistance. The National Health Service intends to provide personalised care; using blood monitoring thresholds modified to an individual's genetics is a rare example in the field of mental health. Duffy genotyping can be requested as part of an extended blood type from the local transfusion laboratory and then performed by the International Blood Group Reference Laboratory in Bristol. A simple referral to haematology might read: ‘*Mr X is an N year old of E ethnicity with no history of spontaneous infection or other serious physical illness. Repeated FBCs have shown neutrophil counts below the normal reference range. An extended blood type reveals the Duffy-null genotype. Other tests including a blood film, antibody screening, myeloma screen, CRP and haematinics are normal. The abdominal ultrasound does not show splenomegaly. Current medication includes: [list]. Is this consistent with BEN and can you confirm this to allow registration with the clozapine monitoring service using the BEN reference ranges?*’ The molecular basis of BEN has only recently been established, and although not necessary for a confirmation of BEN, it does add weight and useful information when individuals continue to show low ANC counts even late in clozapine treatment. The Royal College of Psychiatrists now recommends consideration of testing for the Duffy-null genotype for those with African ancestry where clozapine is being considered or used.^39,40^ It is also possible to review a patient who has had clozapine discontinued when used with the normal thresholds if a finding of BEN is subsequently made, with clozapine restarting on licence. A variety of approaches have been taken to restarting clozapine, including the use of lithium or G-CSF to support neutrophil counts.^41^ However, given the distinctions between CIN and CIA, the rationale for these interventions is seriously weakened. They involve manipulation of neutrophil counts which are mostly likely to be non-pathological and clinically insignificant, using drugs that are potentially toxic in both the short and long term, purely for the benefit of the monitoring system, rather than the patient; in other words, ‘treating the test’. A simpler (and safer) approach would be to re-examine the nature of the problem results and then, if appropriate, initiating or reinitiating using the US criteria. This approach has been reported in a significant UK sample. When a 19-year cohort of 3731 UK CNRD cases was examined, only 566 (15%) were found to have met the US thresholds for discontinuation. The same group had found that in their trust, 115 individuals had been placed on the CNRD in 17 years, but only seven (6%) would have met the US criteria.^42^ When 62 of these patients were rechallenged using off-licence agreements to use the US thresholds, 59 (95%) were successfully treated, and of the three placed back on the CNRD, only one would have had to stop had the US criteria been used.^43^ This suggests that the US thresholds increase access to clozapine with little or no change in safety. Although this approach is not yet widespread in the UK, all the monitoring services have processes and application forms for off-licence use with altered blood monitoring parameters. A systemic improvement would be for the UK licence to use thresholds aligned with those used safely in the US and, given the limited evidence for and irrationality of prolonged monitoring, reconsider the duration and frequency of monitoring. This would require the licence holders to apply to the MHRA.^44^

It is always useful to listen to what patients and their families have to say. Most psychiatrists will be surprised by this comment from a carer about clozapine discontinuation following a single red result late in treatment, resulting in a prolonged relapse, which they then discovered had been unnecessary, not only on the basis of the current scientific evidence but even through the licence. If a repeat sample had been taken promptly – ideally in the afternoon after some exercise – then clozapine could have been continued: ‘*The problem we had with it was that nobody did anything at all and haven't done in six months to determine the low count, nothing whatsoever … Lack of evidence and overwhelming data to suggest that low count had nothing to do with clozapine, but we were given the red (below threshold neutrophil) results and were simple told on a Friday night we had to stop clozapine.*’^7^ Stopping clozapine is a high-stakes decision, requiring consideration of the risks of continuation; these may be limited compared with the benefits, which for many are great, including reductions in all-cause mortality and specifically mortality from suicide.^45^

## Key points

- CIA is an idiosyncratic, dose-independent drug reaction which occurs early in treatment and is characterised by rapid and profound drops in neutrophils.
- As the duration of treatment increases, episodes of neutropenia without these characteristics become increasingly unlikely to be serious or clozapine related.
- The most recent evidence suggests that neutropenia, as opposed to agranulocytosis, is no more common with clozapine than with other antipsychotics.
- Patients from minority groups experience significant disadvantages regarding access to clozapine. Knowledge of BEN and how to identify it and use specific within-licence monitoring may improve access.
- In 2015, the US FDA approved significantly lower but equally safe ANC thresholds. If these were used in the UK, many individuals might benefit from both improved access to clozapine and avoidance of unnecessary discontinuations.
- Over 80% of those prohibited from clozapine using the UK criteria could resume clozapine treatment using the US thresholds and without the use of lithium or G-CSF. However, this does not include cases of agranulocytosis (ANC <0.5 × 10^−9^ /L).
- All the UK clozapine providers have systems in place to use alternative blood monitoring parameters.

## Data Availability

Data availability is not applicable to this article as no new data were created or analysed in this study.

## About the authors

**Edward Silva** is a consultant forensic psychiatrist at Mersey Care NHS Foundation Trust, Rathbone LSU, Rathbone Hospital, Liverpool, UK. **Sophie Legge** is a research associate at the Centre for Neuropsychiatric Genetics and Genomics, Division of Psychological Medicine and Clinical Neurosciences, School of Medicine, Cardiff University, Cardiff, UK. **Cecilia Casetta** is a consultant psychiatrist at South London and Maudsley NHS Foundation Trust, London, UK; and a clinical lecturer at the Institute of Psychiatry, Psychology & Neuroscience, King's College London, London, UK. **Eromona Whiskey** is a consultant pharmacist at South London and Maudsley NHS Foundation Trust, London, UK; and an honorary senior lecturer at the Institute of Pharmaceutical Science, King's College London, London, UK. **Ebenezer Oloyede** is a principal pharmacist at South London and Maudsley NHS Foundation Trust, London, UK; and a researcher at the Department of Psychiatry, University of Oxford, Warneford Hospital, Oxford, UK. **Siobhan Gee** is a consultant pharmacist at South London and Maudsley NHS Foundation Trust, London, UK; and an honorary senior lecturer at the Faculty of Life Sciences and Medicine, King's College London, London, UK
